# The influence of socioeconomic environment on the effectiveness of alcohol prevention among European students: a cluster randomized controlled trial

**DOI:** 10.1186/1471-2458-11-312

**Published:** 2011-05-13

**Authors:** Maria Paola Caria, Fabrizio Faggiano, Rino Bellocco, Maria Rosaria Galanti

**Affiliations:** 1 Department of Public Health Sciences, Karolinska Institutet, Norrbacka, SE-171 76 Stockholm, Sweden; 2 Department of Clinical and Experimental Medicine, Avogadro University, Via Solaroli 17, 28100 Novara, Italy; 3 Piedmont Centre for Drug Addiction Epidemiology, Via Sabaudia 164, 10095 Grugliasco, Turin, Italy; 4 Department of Statistics, University of Milano-Bicocca, Via Bicocca degli Arcimboldi 8, U7, 20126 Milan, Italy; 5 Department of Medical Epidemiology and Biostatistics, Karolinska Institutet, Nobelv. 12A, SE 171-77 Stockholm, Sweden

## Abstract

**Background:**

Although social environments may influence alcohol-related behaviours in youth, the relationship between neighbourhood socioeconomic context and effectiveness of school-based prevention against underage drinking has been insufficiently investigated. We study whether the social environment affects the impact of a new school-based prevention programme on alcohol use among European students.

**Methods:**

During the school year 2004-2005, 7079 students 12-14 years of age from 143 schools in nine European centres participated in this cluster randomised controlled trial. Schools were randomly assigned to either control or a 12-session standardised curriculum based on the comprehensive social influence model. Randomisation was blocked within socioeconomic levels of the school environment. Alcohol use and alcohol-related problem behaviours were investigated through a self-completed anonymous questionnaire at baseline and 18 months thereafter. Data were analysed using multilevel models, separately by socioeconomic level.

**Results:**

At baseline, adolescents in schools of low socioeconomic level were more likely to report problem drinking than other students. Participation in the programme was associated in this group with a decreased odds of reporting episodes of drunkenness (OR = 0.60, 95% CI = 0.44-0.83), intention to get drunk (OR = 0.60, 95% CI = 0.45-0.79), and marginally alcohol-related problem behaviours (OR = 0.70, 95% CI = 0.46-1.06). No significant programme's effects emerged for students in schools of medium or high socioeconomic level. Effects on frequency of alcohol consumption were also stronger among students in disadvantaged schools, although the estimates did not attain statistical significance in any subgroup.

**Conclusions:**

It is plausible that comprehensive social influence programmes have a more favourable effect on problematic drinking among students in underprivileged social environments.

**Trial registration:**

ISRCTN: ISRCTN18092805

## Background

Alcohol use is a major cause of mortality and morbidity among young people, being implicated in large proportions of unintentional injuries [1-4], as well as of violent behaviours resulting in homicides and suicides [5,6]. Underage alcohol drinking has been also associated to school drop-out [7], and unsafe sex [3], which in their turn predict poor general health later in life [8]. Studies in the United States, Australia, and Europe have indicated that early onset of alcohol use is a predictor of substance abuse and alcohol dependence in adulthood [9-11]. Although most of these behaviours are associated with socioeconomic characteristics among youths [12], little evidence exists in the literature in support of a socioeconomic gradient of alcohol use during adolescence [13]. However, some differences emerge when investigating different drinking dimensions. Some studies among young people have reported a direct relationship between household income and frequency of alcohol consumption [14,15], but an inverse relationship between occupational level of the father and quantity of alcohol consumed on a typical drinking occasion [16]. Also, other studies suggested that low socioeconomic status may be associated with problematic drinking in youth [17-19].

Given social differences in profiles of alcohol use and the recognized need to reduce the social gap in the burden of risk factors [20], an evaluation of preventive programmes across social strata is desirable. Since most preventive programmes are delivered at the community level (e.g. in schools) rather than at the individual level, measures of social disadvantage should be assessed accordingly, at the collective level. In fact, recent studies in the United States reported complex associations between community-level indicators of socioeconomic status and underage drinking [21-23]. Besides, research has shown that neighbourhood socioeconomic position influences health related behaviours [24,25]. Several potential mechanisms have been hypothesized such as availability of health, social and community support services and provision of tangible support (e.g. transportation, leisure and sporting facilities) [26]. Therefore, it is plausible that the context into which a preventive program is brought will influence its effectiveness.

However, this effect has rarely been considered in the evaluation of school-based interventions against alcohol use, for instance comparing intervention's effects between areas with different social level.

The purpose of the present study was to analyse whether the social environment at the level of the school area affects the effectiveness of preventive school curricula on alcohol use. The EU-Dap (EUropean Drug Abuse Prevention) study was the first European trial designed to evaluate the effectiveness of a new school-based programme ("Unplugged") for substance use prevention. Participation in the programme was associated with a lower occurrence of episodes of drunkenness and alcohol-related behavioural problems 18 months after baseline, compared to usual curricula, while average alcohol consumption was not impacted [27,28].

Since the socio economic status of the living environment has been associated with adolescents' educational achievements and health behavior [29,30], we hypothesized a different preventive impact of the intervention in environments with different socio-economic level.

## Methods

The EU-DAP trial (ISRCTN-18092805) took place simultaneously in nine centres of seven European countries: Austria, Belgium, Germany, Greece, Italy, Spain and Sweden. The research protocol complied with the ethical requirements foreseen at the respective study centres.

### Experimental Design and Sample

The study was a cluster randomised controlled trial among students attending junior high school in the participating regional centres: one urban community from each involved country (the municipality of Wien, Merelbeke, Kiel, Bilbao, the North-west region of Thessaloniki, and the Stockholm region of Sweden) and three urban communities from Italy (the municipality of Turin, Novara, and L'Aquila). One-hundred and seventy schools were selected on the basis of inclusion criteria and of willingness to cooperate.

Schools were sampled in order to achieve a balanced representation of the underlying average socioeconomic status of the population in the corresponding catchment area. Prior to randomisation schools within each regional centre were ranked by social status indicators and classified, as schools of either high, medium, or low socioeconomic level on the basis of tertiles of the corresponding distribution. This stratification was done independently by each regional centre using the most reliable and recently available data. Different indicators were used (Table 1). Indicators of population's social conditions of the catchment area of the school were used in Greece and Sweden. In Germany, Belgium and in the two Italian centres of Turin and Novara type of school was used, because there is a clear social class gradient in the corresponding school systems. In the remaining regional centres a combination of area and school indicators was used.

**Table 1:** Indicator of social status, number of enrolled schools and students at baseline, by regional centre

Regional centre	Indicator of socio economic level	Number of schools					
		Socio economic level of the school area					
		Low		Medium		High	
		Program	Control	Program	Control	Program	Control
Austria - Wien	Average income in the school district, type of school and proportion of immigrant children in the school.	1 (68)	3 (168)	2 (84)	3 (269)	3 (165)	2 (104)
Belgium - Merelbeke	Type of school.	3 (211)	2 (50)	1 (47)	3 (126)	2 (134)	2 (141)
Germany - Kiel	Type of school.	3 (112)	2(57)	3 (142)	2 (75)	3 (122)	2 (84)
Greece - Thessaloniki	Indicators of social conditions of the school area	3 (117)	2 (97)	4 (189)	3 (147)	3 (92)	2 (90)
Spain - Bilbao	Unemployment rate, indicators of socioeconomic development in the region, type of school.	2 (90)	2 (63)	1 (27)	2 (80)	1 (78)	2 (91)
Sweden - Stockholm	Unemployment rate, proportion of residents with compulsory or lower education, proportion of residents on social welfare in the region.	6 (273)	2 (151)	4 (157)	3 (136)	4 (147)	4 (169)
Italy - L'Aquila	Proportion of immigrant residents in the school area, proportion of students of the school with at least one parent with college education.	2 (82)	3 (108)	2 (81)	2 (96)	3 (137)	1 (46)
Italy - Novara	Type of school.	2 (79)	3 (190)	2 (82)	0 (0)	2 (129)	1 (36)
Italy - Turin	Type of school.	5 (205)	4 (336)	5 (234)	4 (271)	6 (263)	4 (351)
Whole sample		27 (1237)	23 (1220)	24 (1043)	22 (1200)	27 (1267)	20 (1112)

Schools in each centre were randomised to either the intervention or a "usual curriculum" (control) group within the socioeconomic stratum.

Students in the intervention group participated in the EU-Dap substance abuse preventive programme consisting of 12 one-hour sessions designed to tackle adolescents' use of alcohol, tobacco and illicit drugs. This new curriculum is based on a Comprehensive Social Influence model [31], and focuses on developing and enhancing interpersonal and intrapersonal skills. Sessions on normative education and information about the harmful health effects of substances are also provided. Details on the curriculum theory base and content have been provided elsewhere [32]. Ordinary classroom teachers were trained during a 3-day course in interactive teaching techniques. Thereafter, they administered the intervention sessions over three months. The protocol of the programme implementation was carefully standardised. Students in the control group received the programme normally in use at their schools, if any.

In October 2004, 7079 students aged 12-14 years (3532 in control schools and 3547 in intervention schools) participated in the pre-test survey. Post-test data were collected in May 2006, i.e. at least 18 months after baseline. Data from baseline and follow-up surveys were matched using a self-generated anonymous code [33], leaving an analytical sample of 5541 students (78.3%). Additional information on the study design and study population has been published elsewhere [34].

### Data collection and measures

Self-reported substance use, along with relevant cognitive, attitudinal, and psychometric variables, was assessed by an anonymous paper-and-pencil questionnaire, administered in the classrooms without teachers' participation. Students were reassured about the confidentiality of their reports and the anonymous code procedure was explained. Apart from language adaptation, the same questionnaire and assessment procedures were used across all countries and all data collection points. Most questions were retrieved in the "Evaluation Instruments Bank" (http://eib.emcdda.europa.eu/), assessed in 2004. A test-retest evaluation of the survey instrument was conducted during a pilot study [33].

The outcomes of interest in the present analysis were: average frequency of current alcohol consumption, past 30-day prevalence of episodes of drunkenness, intention to drink and to get drunk within the next year, and occurrence of problem-behaviours related to the use of alcohol. The latter occurrence was assessed by asking the students whether they, in the past 12 months, had experienced any of 11 problems, including fighting and injury, because of their drinking. Intentions to drink alcohol or to get drunk within the next year were reported by the students on a 4-point scale ranging from "Very likely" (1) to "Very unlikely" (4). In addition, we explored some individual psycho-social characteristics: perceived school performance, exposure to siblings' alcohol use, and perceived parents' tolerance concerning alcohol drinking. The questions used for the assessment of outcomes and predictors have been fully described in previous reports [34,35].

We dichotomized the frequency of alcohol consumption into "Any current drinking" versus "No current drinking" as well as into an indicator of frequent drinking ("Drinking at least weekly" versus "Drinking less than weekly or not at all"). Also intentions to drink and to get drunk were dichotomized in "Very likely" or "Likely" versus "Unlikely" or "Very unlikely". Since the baseline prevalence of each alcohol-related behavioural problem and of episodes of drunkenness was very low, we collapsed these responses into two dichotomous outcomes of "No alcohol-related problems" versus "Any problem" in the past 12 months, and "No episodes of drunkenness" versus "Any episode" in the past 30 days respectively. Perceived school performance, based on self-comparison of own grades with those of the classmates, was coded as "Worse" versus "As good or better". Exposure to siblings' alcohol use was dichotomized, and students without siblings were considered unexposed to this influence. Perceived parents' tolerance concerning alcohol drinking was dichotomized into "Would not allow me to drink at all" versus "Others". Assessed socio-demographic characteristics included gender, age, school-grade and the family living situation coded as "Living with both parents" versus "Other living situation".

### Statistical analysis

We performed descriptive statistical analyses to summarize the main characteristics of the study sample. We tested the baseline equivalence by experimental condition of outcomes and predictors of interest separately by socioeconomic level using chi-square tests with the appropriate degrees of freedom.

Odds Ratios (OR) and their corresponding Confidence Intervals (95% CI) were estimated as measure of association between experimental conditions and behavioural outcomes, separately for each socioeconomic level of the school area. A multilevel logistic regression model was fitted to account for the hierarchical structure of the data with one random effect at the classroom level and one at the regional centre level [36]. We tested several established predictors of substance use as potential confounding variables. These included gender, age, family living situation, family alcohol use, perceived school performance, perceived parents' tolerance concerning alcohol drinking, and the baseline status of the behaviour under study. Models were adjusted for variables on which the intervention and control group significantly differed at baseline and for the baseline status of the outcome. We also formally tested for statistical interaction by including in the regression model a cross-product term between the treatment condition and the socioeconomic status indicator, coded in dummy variables. A significant test statistic based on the likelihood ratio test for this interaction term is evidence that treatment effects vary by school socioeconomic level. All analyses were performed using the statistical package MLwiN 2.2 [37]. All outcome analyses were intent-to-treat.

## Results

The sample consisted of 5541 students, 49.1% of whom were females. Mean age was 13.2 years. At baseline, gender and age distributions differed among social levels (data not shown). Schools in the lowest level had a higher percentage of male and older students. Students in schools of high socioeconomic level were more likely than students in other schools to drink at least monthly (17.2% vs. 14.6%, p = 0.01) and to have intention to drink (43.7% vs. 39.0%, p < 0.01) while students in schools of low socioeconomic level were more likely to report recent episodes of drunkenness (7.0% vs. 4.0%, p < 0.01), intention to get drunk (20.0% vs. 17.6%, p = 0.03), and alcohol-related problem behaviours (4.2% vs. 3.0%, p = 0.02). The only difference among social levels with regard to the considered psychosocial variables was that students in schools of low socioeconomic level compared to other adolescents were more likely to perceive their school performance as worse than average (10.7% vs. 6.1%, p < 0.01).

**Figure 1:**
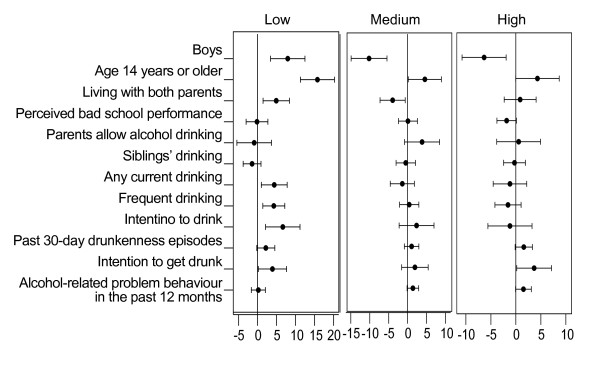
**Differences in baseline characteristics between control and intervention condition by socioeconomic level of school area**. Differences in prevalence and confidence interval between control and intervention condition of baseline characteristics of the study sample, by socioeconomic level of the school area: the EU-Dap Study 2004-2006. A difference above zero means that the prevalence is higher among controls, below zero means that the prevalence is higher among students in the intervention arm.

Figure 1 shows the sample size and the equivalence of some baseline characteristics by experimental condition, separately by socioeconomic level. Within levels of socioeconomic environment we found different distributions between the control and intervention groups for gender, age, family living situation, frequency of alcohol consumption, and intentions to drink and to get drunk. Controls in the lower social level had higher proportions of well-known predictors of alcohol use (male gender, older age and early drinking experience) compared to students in the intervention group.

Missing information at baseline was negligible for any of the assessed characteristic (at the most 2.1%, data not shown).

Participation in the programme was associated with a significantly lower prevalence of episodes of drunkenness and of intention to get drunk, compared to usual curricula, among students attending schools in low socioeconomic context (Table 2). For both outcomes the estimated OR was approximately 0.60.. The same students had an OR of 0.68 of reporting behavioural problems due to their drinking, but this effect was only marginally significant (p = 0.06). Concerning the frequency of alcohol consumption, the estimated effects did not reach statistical significance within sub-groups, but the estimates were consistently lower among students attending schools in disadvantaged contexts. No significant programme's effects emerged for students in schools of medium or high socioeconomic level. Interactions between intervention condition and socioeconomic status at the area level were found to be statistically significant only for intention to get drunk (p = 0.02).

**Table 2:** Programme effects by socioeconomic level of the school area from multilevel models

Socioeconomic level of the school area								
	Low		Medium		High		Whole sample	
	(n = 1819)		(n = 1742)		(n = 1980)		(n = 5541)	
	OR	95%CI	OR	95%CI	OR	95%CI	OR	95%CI
**Any current drinking**	0.84	0.64-1.09	1.08	0.77-1.52	0.93	0.69-1.24	0.95	0.81-1.12
**Weekly drinking**	0.83	0.61-1.12	1.14	0.82-1.58	0.91	0.69-1.21	0.92	0.77-1.09
**Intention to drink in the next year**	0.76	0.58-1.00	1.12	0.83-1.50	1.18	0.91-1.53	0.99	0.85-1.16
**Episodes of drunkenness in the past 30 days**	0.63	0.47-0.88	0.92	0.65-1.31	0.88	0.62-1.23	0.79	0.65-0.95
**Intention to get drunk in the next year**	0.61	0.48-0.79	1.00	0.75-1.32	0.96	0.73-1.26	0.82	0.71-0.96
**Alcohol-related problem behaviour in the past 12 months**	0.68	0.44-1.06	0.97	0.63-1.49	0.85	0.58-1.25	0.78	0.62-0.98

Results from multilevel models adjusted for gender, age, family living situation and baseline status of the outcome: odds ratios (OR) and 95% confidence interval (95%CI) of alcohol-related behaviour for students in the intervention group compared to the controls, by socioeconomic level of the school area. The EU-Dap Study, 18-month follow-up.

## Discussion

In a multi-centre trial among European students we found some evidence that the effectiveness of a comprehensive social influence school-based preventive programme on problematic drinking might differ by socioeconomic environment of the school.

The differences indicated a higher preventive impact of the curriculum on episodes of drunkenness and intention to get drunk among students attending schools in a socially deprived context, compared to students in medium or high social context. The effects of the programme on the frequency of alcohol consumption and the intention to drink were weak and not statistically significant in subgroups, in line with results on the whole study sample [28]. However, even for these outcomes the direction of the estimated effects suggested a higher impact of the curriculum in schools in low social context.

The absence of statistical significance in most interaction tests is compatible with homogeneity of the effects among social strata. However, given the overall pattern of associations, consistently indicating the most favourable effect in areas with low social index, it is also plausible that an existing difference was not detected due to limitations of the study, in particular the imperfect classification of social status of the living environment and the limited sample sizes. Few studies have examined how socioeconomic characteristics influence the effectiveness of substance use school-based prevention. If only life skills training approaches are considered, the evidence is extremely scant and based on observations limited to low social class contexts. In fact, evaluation studies have reported preventive effects on alcohol use in low socioeconomic contexts for Botvin's "LifeSkills Training" [38-40] as well as for the "keepin' it REAL" curriculum of the Drug Resistance Strategies Project [41]. None of these studies provided a comparison of the programme impact with upper social context populations. As an exception, the original edition of Project ALERT was proven equally effective in schools with populations of high and low average social level, but the programme resulted in only short lived effects for alcohol use [42].

To our knowledge, only one recent study has investigated how neighbourhoods influence the effectiveness of a school curriculum in preventing alcohol use [43]. This study reported that living in poorer neighbourhoods decreased the programme's effectiveness in one ethnic subgroup of the sample.

A possible explanation for the indication of an effect modification of social environment on problematic drinking in our study is that the curriculum was more relevant to schools with average low socioeconomic status of the population. It is also plausible that neighbourhood disadvantage correlates with lack of educational resources and of social and familial support to adolescents. Therefore, the relative "preventive gain" from school prevention would be higher in these under-privileged contexts.

Differential teacher's response to training is another possible explanation. Teachers in schools from socially disadvantaged communities may have taken a greater advantage of the training, improving their capability to conduct interactive teaching to a larger extent than teachers in communities of medium or high socioeconomic status. It is also possible that contamination occurred to a larger extent in control schools from medium or high socioeconomic areas, if these schools conducted other health promoting interventions, based on skill-enhancing methods similar to the "Unplugged" curriculum.

There are three major weaknesses in this study. First, the sample size was calculated to study the programme effects on the whole sample. Economic and organizational difficulties made it impossible to sample a number of schools sufficient to explore differential effects across sub-groups. Given the need to employ a multi-level analysis, the study had limited statistical power to detect intervention effects for specific subgroups. Despite the lack of power, tendencies in the results were consistent in indicating a higher effectiveness in socially disadvantaged contexts, with significant interaction for one outcome.

Secondly, the participating centres classified the socioeconomic status of school areas using the best locally available indicators and sources of information, that were however different among centres and not validated. This may have lead to measurement error and misclassification of social grouping for some schools. However, since schools in the EU-Dap study were randomised within blocks of social level the misclassification would be independent from the experimental arm as well as from the study outcome. The most probable consequence of this unconditional classification error would be to bias the effect estimates towards the value expected under the null hypothesis, i.e. an underestimation of the effect modification.

Thirdly, intervention and control arms within each socioeconomic level differed on some potential confounders related to baseline characteristics, despite the random assignment. This was probably due to group allocation of a relatively low number of schools within each socioeconomic block. Therefore, some residual confounding within strata could be present, although all analyses were adjusted for measured baseline factors that could constitute potential confounders.

Lack of information on socioeconomic status at the individual level could also be acknowledged as a limitation. However, this was rather the consequence of a deliberated choice, since children's reports of parental occupation or education are generally not reliable [44].

This was one of the few studies designed to consider differential effects of a preventive programme across socioeconomic groups. In fact, the assignment of schools to treatment or control conditions was accomplished through block randomisation that controlled for environmental socioeconomic characteristic, thus achieving a balanced representation of social strata in the study sample. Also, many different behavioural aspects of alcohol use were investigated.

## Conclusions

The innovative school curriculum evaluated in the EU-DAP study seems to have a beneficial preventive effect on problem drinking, motivating its further dissemination in schools in lower socioeconomic levels.

Since higher prevalence rates of unhealthy behaviours among lower socioeconomic groups contribute substantially to socioeconomic inequalities in health [45], universal prevention programmes that are effective in lower socioeconomic groups may be useful in reducing this socioeconomic gap, one of the major priorities of public health policy in Europe.

## Competing interests

The authors declare that they have no competing interests.

## Authors' contributions

FF and MRG designed the study. MPC and MRG drafted the paper. FF and RB contributed to revising the paper. MPC performed the statistical analysis. The members of the EU-Dap Study Group carried out the intervention and collected the data. MPC has overall responsibility for the paper. All authors contributed to and approved the final manuscript.

## Pre-publication history

The pre-publication history for this paper can be accessed here:

http://www.biomedcentral.com/1471-2458/11/312/prepub
